# Oilseed By-Products Valorization Using Lactic Acid Fermentation: Nutritional and Technological Aspects of Applications in Wheat Bread

**DOI:** 10.3390/molecules31010015

**Published:** 2025-12-20

**Authors:** Jakub Roman Królak, Agnieszka Makowska, Katarzyna Waszkowiak, Kamila Myszka, Kinga Stuper-Szablewska, Anna Przybylska-Balcerek, Katarzyna Rzyska-Szczupak

**Affiliations:** 1 Department of Food Technology of Plant Origin, Faculty of Food Science and Nutrition, Poznań University of Life Sciences, Wojska Polskiego 31, 60-624 Poznan, Poland; jakub.krolak@up.poznan.pl; 2 Department of Gastronomy Science and Functional Foods, Faculty of Food Science and Nutrition, Poznań University of Life Sciences, Wojska Polskiego 31, 60-624 Poznan, Poland; 3 Department of Biotechnology and Food Microbiology, Faculty of Food Science and Nutrition, Poznań University of Life Sciences, Wojska Polskiego 48, 60-637 Poznan, Poland; kamila.myszka@up.poznan.pl; 4 Department of Chemistry, Faculty of Forestry and Wood Technology, Poznań University of Life Sciences, Wojska Polskiego 75, 60-625 Poznań, Poland; kinga.stuper@up.poznan.pl (K.S.-S.); anna.przybylska@up.poznan.pl (A.P.-B.); katarzyna.rzyska@up.poznan.pl (K.R.-S.)

**Keywords:** oilseed cake, *Lactobacillus*, bioactive compounds, wheat bread

## Abstract

This study aimed to determine the effect of lactic acid fermentation with *Lactiplantibacillus plantarum* on the bioactive compound composition and fatty acid profile of black cumin, camelina, milk thistle, and evening primrose cakes, as well as to evaluate their application as ingredients in wheat bread production (9% of wheat flour substitution). Fermentation increased the content of flavonoids and phenolic acids in camelina cake by approximately 30%, while causing a 30% decrease in carotenoid content. In black cumin cake, an eightfold increase in 4-hydroxybenzoic acid content and a 10% reduction in thymoquinone were observed. For milk thistle, silymarin content decreased by approximately 10%. Fermentation increased the proportion of saturated fatty acids (SFAs) and monounsaturated fatty acids (MUFAs), reducing polyunsaturated fatty acids (PUFAs) in all analyzed cakes. Breads containing 9% fermented cakes exhibited lower specific volume and greater hardness (22–80%), gumminess (17–64%), and chewiness (8–48%), compared to the breads with unfermented cakes. The contents of bioactive compounds in breads depended on the type of cake added. The bread with fermented camelina cake showed a 15% increase in flavonoid content and higher levels of selected phenolic acids compared to the bread with unfermented camelina. The breads containing camelina cake, both fermented and unfermented, also had the most favorable physical quality (texture and volume). The amount of ferulic acid in all samples of bread with the addition of fermented cakes was lower in comparison to the bread samples with unfermented cakes.

## 1. Introduction

The escalating global population and increasing demand for food necessitate sustainable food production practices and rational utilization of resources, particularly within the agri-food sector [1]. A significant challenge in modern agriculture is the management of agro-industrial by-products, which often represent underutilized resources [2,3].

Several oilseed crops are cultivated worldwide for their valuable oils, and their residual cakes possess distinct nutritional compositions and bioactive compound profiles. Among the oil plants camelina, black cumin, evening primrose, and milk thistle can be mentioned. Their production, according to FAOSTAT is approximately 100,000 tons, 18,000 tons, 16,000–19,000 tons, and 1900 tons per year, respectively [4]. Oilseed cakes, the solid residues remaining after oil pressing from oilseeds, exemplify by-products with considerable potential for valorization into high-value ingredients for both food and pharmaceutical applications [1,2]. Their use aligns with the principles of circular economy, reducing waste and contributing to environmental sustainability.

Oilseed cakes are rich in macro- and micronutrients, making them promising sources of functional ingredients. Generally, these cakes contain a high amount of protein, ranging from 19.4% to 62.3%, and significant dietary fiber content (6.5% to 37.0%), along with residual oil (8.9% to 36.2%) that contributes energy to diets [5]. They also harbor natural antioxidant compounds, vitamins, and minerals. Camelina press cake contains 35–40% crude protein with essential amino acids like lysine, methionine, and cysteine. Its residual oil is rich in α-linolenic and linoleic acids. It also provides phytochemicals (phenolic compounds, carotenoids, phytosterols, and vitamins), glucosinolates (22.9–46.1 µmol/g), quercetin glycosides, and mucilage, which can act as a prebiotic [6]. Black cumin seed cake is a good source of protein, thymoquinone, and is known for its powerful antioxidant activity [7]. Milk thistle oilseed cake is rich in protein (defatted flour can reach 38.76–44.92% dry mass) and contains significant amounts of linoleic (54.3–57.2%) and oleic acids (25.5–28.1%). Its primary bioactive compounds are flavonolignans, collectively known as silymarin (e.g., silibinin, silychristin, silidianin, and isosilybin), which contribute to its antioxidant potential [8].

The utilization of oilseed cakes in food production represents a strategic approach to enhance nutritional value, reduce waste, and improve resource efficiency within the food industry [9]. Traditionally, most oilseed cakes are used as animal feed or organic fertilizers [1]. However, their rich nutritional profile makes them ideal for direct human consumption, particularly as ingredients of functional foods [3]. Bakery products, being staple foods globally, offer an excellent vehicle for fortification with oilseed cakes to improve their nutritional and health-promoting characteristics. Numerous studies have explored the incorporation of oilseed cakes into bread and other bakery items. Camelina cake has been proposed for use in buns and sandwiches [10]. Ground milk thistle cake has also been used to fortify bread and biscuits, increasing their fat content (with beneficial unsaturated fatty acids) and anti-radical activity, while surprisingly lowering the caloric value due to lower carbohydrate content compared to wheat flour [11]. However, the addition of oilseed cakes, especially at higher concentrations (above 5% for flaxseed and lupine cakes), can negatively impact bread volume and increase hardness, gumminess, and chewiness, and these effects are largely attributed to interference with gluten network formation and high water absorption by fiber [12].

Lactic acid fermentation can offer a promising solution that overcomes the aforementioned technological challenges and additionally improves the nutritional and functional properties of oilseed cakes. Fermentation process can improve the organoleptic attributes and nutritional value of foods, extend their shelf life, and limit antinutritional factors [6]. For example, fermenting flaxseed cake with *Rhizopus oligosporus* or *Lactobacillus plantarum* significantly increases its antioxidant potential, enhances phenolic compound release, and improves radical scavenging activity [13]. Lactic acid fermentation of flaxseed cake has also been shown to reduce undesirable changes in bread texture, resulting in lower hardness, gumminess, and chewiness, and improving overall consumer acceptability [12]. Similarly, fermenting camelina press cake with yogurt starter cultures boosts beneficial lactic acid bacteria (LAB) viability (exceeding 10^10^ CFU/g), increases free amino acids and polyphenolics, and demonstrates good antioxidant potential. The presence of mucilage and essential amino acids in camelina cake further supports LAB growth, making it an excellent substrate for fermentation [6]. Furthermore, fermentation has the potential to degrade antinutritional compounds present in oilseed cakes, for example, cyanogenic glycosides in flaxseed cake [14,15].

Despite the growing interest in using different kinds of oilseed cakes in bakery, comprehensive studies on the combined effects of adding unfermented and fermented oilseed cakes on the physical characteristics and bioactive compound content of wheat bread are still limited.

This study aims to determine the effect of black cumin, camelina, milk thistle, and evening primrose cake lactic fermentation with *Lactiplantibacillus plantarum* on bioactive components and fatty acid compositions of these cakes, as well as their application as ingredients in wheat bread production (9% substitution of wheat flour). The analysis of bread physical properties (i.e., texture, volume, and color) was included in the study.

## 2. Results

### 2.1. Bioactive Compounds in Unfermented and Fermented Oilseed Cakes

The unfermented (UF) and fermented (F) cakes were analyzed for flavonoids, phenolic acids, and compounds characteristic of individual cake types, such as thymoquinone (black cumin cake), silymarins (milk thistle cake), and carotenoids (camelina cake). The results are shown in Table 1. Due to the lack of evening primrose cake fermentation (see Section 3 ), the results only for unfermented evening primrose cake were reported. The lack of fermentation in evening primrose cake was probably due to the presence of polyphenolic compounds. Peschel et al. [16] demonstrated significantly higher total phenolic content and antioxidant activity of evening primrose cake compared to other oilseed cakes. Furthermore, Miedzianka et al. [17] reported that phenolic compounds of evening primrose (i.e., numerous phenolic acids, gallic acid derivatives, and tannins) also exhibited antibacterial activity.

It was shown that camelina cakes were the richest source of flavonoids among all analyzed cakes. Eight different compounds were identified with a total content of 36.14 mg/kg d.m. Among the compounds identified in camelina cakes, apigenin was the most abundant (approximately 30% of the total flavonoid content), followed by quercetin and rutin. Flavonoids such as luteolin, vitexin, and naringenin occurred at approximately 4 mg/kg, while catechin was the least abundant. In the camelina cake, according to the literature [18,19], other flavonoids were also identified, such as quercetin 3-O-glucoside, kaempferol glycosides, as well as 4-vinylphenol, 4-vinylsyringol, and 4-vinylguaiacol. These compounds were not stated in our study. As a result of fermentation, the flavonoid content in the camelina cake extracts increased by approximately 30%, compared to the unfermented one, and reached 46.94 mg/kg d.m. In case of all substances the increase was similar.

Seven different flavonoids (catechin, kaempferol, luteolin, naringenin, quercetin, rutin, and vitexin) were identified in the evening primrose cakes. Their total content was 12.58 mg/kg d.m., which was 60% lower than in camelina cake. In evening primrose cake, vitexin and rutin were the most abundant flavonoids. Apart from these substances Kiss et al. [20] also identified epicatechin, procyanidins (B1, B2), pentagalloylglucose, and myricetin.

Small amounts of flavonoids were also determined in milk thistle cakes (0.13 mg/kg d.m.). In this cake, only rutin and vitexin were identified. There were no differences between identified flavonoid contents in the unfermented and fermented milk thistle cake.

There were no identified flavonoids in the black cumin cake in this study, but there are some reports in the literature on the presence of kaempferol (kaempferol) and *p*-coumaroyl derivatives in it (determined using LC–Q–TOF–MS/MS), as well as quercetin and trace amounts of its glycosides [21].

The camelina cake also contained the highest concentrations of phenolic acids. It contained a total of 1149.60 mg/kg d.m. of phenolic acids. More than half of this amount was sinapic acid (652.22 mg/kg), followed by caffeic acid (132.70 mg/kg d.m.) and cinnamic acid (132.97 mg/kg d.m.). Ferulic, chlorogenic, 4-hydroxybenzoic, syringic, and *p*-coumaric acids were also present in smaller amounts. In the fermented cakes the amount of phenolic acids increased by approximately 30%, compared to the unfermented ones. The content of phenolic acids in the remaining cakes was significantly lower. Evening primrose cake contained over ten times less phenolic acids than camelina (93.75 mg/kg d.m.). Among the identified acids (gallic, ferulic, *p*-coumaric, caffeic, and chlorogenic), gallic acid was the most abundant (63.40 mg/kg d.m.). Low concentrations of phenolic acids were identified in the black cumin cake and milk thistle cake (0.19 and 0.16 mg/kg d.m., respectively). Hydroxybenzoic acid and *p*-coumaric acid were detected in the black cumin cake, while *p*-coumaric and chlorogenic acids were stated in the milk thistle cake. After fermentation the amount of 4-hydroxybenzoic acid in extract from the black cumin cake increased eightfold. There was no difference in phenolic acid content in the unfermented and fermented milk thistle cakes.

Thymoquinone is a characteristic compound found in black cumin. Its content in the unfermented black cumin cake was 1150 mg/kg d.m., and its amount decreased 10% as the result of fermentation. In the milk thistle cake, the dominant compounds are the following flavonolignans: silymarin, silybin, and silicristin. Their content, determined as the sum of these compounds, was 30.53 mg/kg d.m. In the fermented cake, their amount decreased by approximately 10%, compared to the unfermented one. Carotenoids were identified in cameline cakes. The presence of β-carotene (30.50 mg/kg d.m.), lutein (3.55 mg/kg d.m.), and zeaxanthin (0.05 mg/kg d.m.) were detected the cakes. After fermentation their amount decreased by 30%.

Based on the literature, it can be assumed that changes in the content of phenolic acids and flavonoids in extracts obtained from cakes due to fermentation could be caused by the activity of the *Lactiplantibacillus plantarum* bacteria enzymes [22]. As a result of their activity, both an increase in the content of the analyzed compounds and, under certain conditions, a decrease in their content could be observed. Feruloylesterase hydrolyzes ester bonds between phenolic acids (e.g., ferulic acid, *p*-coumaric acid) and polysaccharides or lignin in the cell walls of plant material. This allows the release of free phenolic acids from their bound form. β-glucosidase, produced by the bacteria *L. plantarum*, degrades O-glycosidic bonds of flavonoids and some phenolic acids, releasing aglycones. This improves the bioavailability of flavonoids and some other polyphenols [23]. Tannase synthesized by bacteria hydrolyzes ester bonds in tannins (especially gallic acid and its derivatives), releasing free phenolic acids [24]. The present phenol decarboxylase decarboxylates hydroxycinnamic phenolic acids (e.g., *p*-coumaric acid), converting them to their vinyl derivatives (e.g., 4-vinylphenol), while vinylphenol reductase reduces vinyl derivatives of phenolic acids, such as 4-vinylphenol, to ethyl derivatives (e.g., 4-ethylphenol), changing the bioactive and sensory properties of the resulting metabolites. Hydroxycinnamate reductase reduces hydroxycinnamic phenolic acids to their corresponding phenylpropionic acids. In the case of *L. plantarum* bacteria, their involvement in the conversion of *p*-coumaric acid to 3-(3-hydroxyphenyl)-propionic acid has been confirmed [22]. Although detailed data on the specific enzymes of *L. plantarum* that influence thymoquinone and silymarin metabolism are lacking, it can be assumed that the existing enzymes with flavonoidase and β-glucosidase activity, as well as microbial oxidoreductase activity, may be involved in the hydrolysis of flavonolignan glycosides and their conversion into aglycones (e.g., silibinin, silidianin) [23].

Previous study also showed that fermentation with *L. plantarum* bacteria can affect the carotenoid compounds in fermented cakes [25]. *Lactiplantibacillus plantarum* has been shown to have the ability to transform carotenoids during fermentation, but these mechanisms are limited and dependent on the strain and environmental conditions. Some *L. plantarum* strains can synthesize their own carotenoids, such as 4,4′-diaponeurosporene (a C30 carotenoid), due to the presence of the crtNM operon (crtM and crtN genes encoding dehydroscualene synthase and dehydroscualene desaturase) [25]. On the other hand, these bacteria can cause partial degradation of β-carotene.

### 2.2. Fatty Acid Profile in Unfermented and Fermented Oilseed Cakes

The fatty acid profiles of the analyzed cakes were typical for these oilseeds (Table 2). The share of saturated fatty acids (SFAs) in the total amount of fatty acids ranged from 22% (black cumin cake) to 7.46% (camelina cake). The share of monounsaturated fatty acids (MUFAs) varied from 36.87% (camelina cake) to 6.70% (evening primrose cake). Polyunsaturated fatty acids (PUFAs) accounted for from 83.7% (evening primrose cake) to 55.65% (camelina cake). Among the PUFAs, linoleic acid (C18:2) was the most abundant. It was the dominant acid in evening primrose cakes (74.9%), milk thistle cakes (68.50%), and black cumin cakes (60.40%). In the case of camelina cakes, α-linolenic acid was the most abundant (36.84%). After fermentation the share of SFA increased. Among the total amount of fatty acids, SFAs constituted 27.03% (22% before fermentation) in black cumin, 11.01% (7.46% before fermentation) in camelina, and 23.38% (19.10% before fermentation) in milk thistle cakes. The share of MUFAs also increased as a result of fermentation. It was found that the percentage share of C16:1 (palmitic acid) and C18:1 (oleic acid) increased in all cakes. Additionally, in the case of camelina cakes the share of C20:1 (eicosenoic acid) and C24:1 (nervonic acid) was higher. The highest proportion of MUFAs was observed in fermented camelina cakes (41.56% of total fatty acids). Fermentation of oilseed cakes reduced the PUFA shares from 60.40 to 52.13 (black cumin cake), from 55.67 to 47.43 (camelina cake), and from 68.50 to 61.70 (milk thistle cake).

The calculated PUFA/SFA ratio (Table 2) expressed the observed changes in fatty acid profile after the oilseed cakes fermentation. For all fermented cakes, the ratio was lower than in the corresponding unfermented samples. A greater decrease in the PUFA/SFA ratio (about half) was observed in the case of fermented camelina cake. The observed changes may be due to lipolytic enzymes secreted by *L. plantarum* bacteria [26].

### 2.3. Physical Characteristics of Bread Containing Unfermented and Fermented Oilseed Cakes

The unfermented and fermented cakes (black cumin, camelina, and milk thistle) were used as a 9% substitution of wheat flour in wheat bread. As mentioned earlier, the evening primrose cakes did not ferment under the applied conditions. Therefore, the experiment did not include an analysis of the effect of these cakes on the nutritional value and characteristics of the bread. The lack of data limits the comparison of bread with evening primrose cakes to breads with the addition of other types of cakes that were fermented.

Physical parameters of the bread, such as specific volume, texture, and color, were determined. The results are presented in Table 3.

Analysis of the textural characteristics of the breads showed that the wheat breads with the addition of cakes were characterized by a significantly higher hardness and chewiness than the wheat bread (control without the additions). This can also be attributed to the high concentration of dietary fiber in the cake. Fiber disrupts the development of the gluten structure, reducing the extensibility of the dough and weakening the gluten network during fermentation. Correlation coefficients were determined between the dietary fiber content of the added cakes and the hardness, chewiness, and gumminess of the bread. The values of these coefficients were 0.784, 0.807, and 0.827, respectively. Although their values were high, the observed correlations were not statistically significant at the 0.05 level. The properties of the analyzed bread depended on the type of cake added. The specific volume of the bread containing unfermented cakes was similar to or greater than that of the control wheat bread. It could be connected with water absorption of the cakes. The cakes contained a large amount of dietary fiber and could absorb large amounts of water. The effect of cake on the volume of bread depends on the amount added. A small amount has been reported to increase the volume of bread, but excessive amounts result in a decrease in the volume of bread [12].

The incorporation of oilseed cakes into bread formulations has gained considerable attention, driven by the desire to enhance the nutritional profile of staple foods with rich sources of protein, fiber, and essential fatty acids [27]. They were often added to gluten-free bread [28]. has been extensive research on using oilseed cakes as additions to bread, including flaxseed, naked pumpkin, milk thistle, sesame, chia, sunflower, almond, walnut, melon seed, and hemp [27,29,30]. This enrichment generally led to significant modifications in the mechanical and physical properties of the final product, notably affecting texture, hardness, and volume. The magnitude and direction of these changes critically depend on the enrichment level, the type of cake, and the method of incorporation. Studies available in the literature have shown that small amounts (2–6%) of additives such as chia, melon seed, or hemp do not affect the volume of bread and may even increase this parameter. In contrast, larger amounts of these additives (10–15%) reduce the volume of bread. Similarly, while small amounts of additives preserve the texture of the bread, larger amounts increase the hardness and other texture characteristics [30,31,32]. A common consequence of incorporating non-wheat components, particularly vegetable proteins and fibers prevalent in oilseed cakes, is a predicted negative effect on bread volume and an increase in product hardness [27].

Analyzing the bread with the addition of fermented cakes, it was found that the bread had a lower specific volume than the bread with the addition of unfermented cakes. It characterized by higher hardness (22–80%), gumminess (17–64%), and chewiness (8–48%). The opposite effect was found in the previous study when analyzing the bread with added fermented flaxseed cakes [12]. The smallest differences in the texture of the bread with unfermented and fermented cakes were noted for the black cumin cakes addition, while the greatest differences were observed for the addition of camelina cakes. Smaller volume and firmer texture of the breads with fermented cakes may result from the impact of a lowered pH environment on the gluten proteins contained in the dough. Lactic acid exerts a complex effect on the structure of the gluten network in bread through several interrelated biochemical mechanisms that fundamentally alter the properties of gluten proteins [27]. Lowering the dough pH activates the natural proteolytic enzymes contained in the flour. The acidic environment creates optimal conditions for endogenous proteases, which initiates the controlled degradation of gluten proteins. These enzymes break down long gluten chains into shorter peptides and amino acids, which causes a “loosening” of the gluten network structure [33]. Lactic acid in the dough affects its rheological properties. This increases the elasticity of the dough while reducing its strength, and the exposure of hydrophobic groups increases the water absorption capacity of the dough. In this experiment, the amount of water added to the dough with the addition of fermented cakes was the same as that of the unfermented cakes’ additions, hence the increased water absorption of fermented cakes resulted in bread with a smaller volume and greater hardness.

The color of the crust of breads with cakes was darker than that of wheat bread and less yellow. The red color saturation of crust of the bread with black cumin cake was lower, while in the case of other samples it was more red. Comparing the color of the bread containing unfermented and fermented cakes it was stated that the crumb of the bread with fermented cakes was lighter, more red, and more yellow than that of the bread with unfermented cakes. The differences can be explained by different structure and porosity of the breads with fermented and unfermented cakes discussed above. They can influence the results of color measurements.

The color of breads with fermented and unfermented oilseed cakes was also characterized using the whiteness index (WI) of the crumb and browning index (BI) of the crust. The WI combines lightness (L*) and the yellow–blue coordinate (b*) into a single value that reflects the overall whiteness of a food product [34]. In the case of bread crumb, a higher WI value indicates a lighter crumb, which is generally preferred by consumers in the case of conventional wheat bread, whereas a decrease in WI is usually perceived unfavorably for this product. For fortified breads, however, a darker color can be acceptable, as it may signal higher nutritional value and the presence of functional components [35]. In this study, the control wheat bread showed the highest crumb WI (66.18). Lower values were noted for the breads containing cake additions. Based on the results, it was stated that the cakes’ fermentation caused a significant increase in the WI value of crumb for the breads with these cake additions. This was observed particularly in the breads with unfermented and fermented black cumin cake (WI values were 30.05 vs. 51.46, respectively) and milk thistle cake (46.03 vs. 53.41, respectively). The cake additions also influenced the change in the bread crust’s BI values. However, the direction of these changes depended on the type of cake. It was found that the BI values for the bread crust with the addition of fermented cake were higher than those for the bread crust with the same unfermented cake. The greatest differences were observed in the case of breads with the addition of camelina cakes. The BI values for bread crust with the addition of unfermented and fermented camelina cakes were 66.78 and 80.99, respectively. The findings are in line with previous reports on the effects of plant-derived additives on bread color parameters, including the study by Dahdah et al. [36], who observed decreases in both BI and WI in breads fortified with olive pomace.

The reduction in WI value of crumb breads with oilseed cakes could be due to the darker color of these raw materials, containing natural pigments (carotenoids, chlorophylls) and phenolic compounds. For example, the darkening of the crumb in bread with black cumin cake could be characteristic of this material, rich in thymoquinone and melanin-type pigments [37]. The increase in the WI value of crumb bread with fermented cakes may be associated with modifications of crumb structure and porosity. Breads with fermented cakes showed lower specific volume and higher hardness (Table 3); this may alter light reflection and influence colorimetric measurements. Lactic fermentation may additionally contribute to a partial degradation of dark pigments [38].

Higher values of the BI of the bread crust with fermented oilseed cakes in comparison to the BI values for bread with the addition of unfermented ones may be caused by more intense browning during baking. Fermentation with *L. plantarum* enhances the content of free amino acids and reducing sugars, which may act as substrates for Maillard reactions. Moreover, lactic acid formed during fermentation may further influence the kinetics of non-enzymatic browning during thermal processing [39].

### 2.4. Bioactive Compounds in Bread Containing Unfermented and Fermented Oilseed Cakes

The contents of bioactive compound in the wheat breads with unfermented and fermented oilseed cakes as well as in the control (the bread without the additions) are presented in Table 4.

A significant increase in flavonoid content (apigenin, luteolin, quercetin, and rutin) was observed in the bread with fermented camelina cake (4.37 mg/kg). It was about 15% higher compared to the bread with the addition of unfermented camelina cake (3.85 mg/kg). No flavonoids were detected in the wheat bread (control) and the bread with black cumin cake. In the breads with unfermented and fermented milk thistle cake additions, only small amounts of vitexin (0.1 mg/kg) were found..

An increase in flavonoid content in sourdough bread with grape pomace and bread with fermented carob flour was also noted in the studies by Torreggiani et al. [40] and Novotni et al. [41]. They found that despite the total flavonoid content in the breads with fermented ingredients being only slightly higher, both their antioxidant activity and profile changed significantly. Their studies showed that more free and more readily available forms appeared, and the proportions between the free phenolic and the bound phenolic acid fractions changed.

Analysis of the phenolic acid content in breads revealed that the dominant acid in all samples was ferulic acid, both in the control bread (121.23 mg/kg) and the bread with cakes (from 89.60 to 130.53 mg/kg). The bread with camelina cakes had the highest phenolic acid content: 236.53 mg/kg (unfermented cake) and 231.25 mg/kg (fermented cake), and a broad spectrum of acids among all breads analyzed in the study. Phenolic acid contents in the breads with the other cakes were lower. The only identified compound in the bread with black cumin cake and the bread with milk thistle cake was ferulic acid, derived from wheat flour.

Based on the obtained results, it was concluded that the fermentation of cakes can both decrease and increase the content of individual phenolic acids in bread made with them. The amount and kind of bioactive compounds which were detected in breads related to their content in the added unfermented and fermented cakes. The bread containing fermented camelina cakes had a higher amount of hydroxybenzoic, caffeic, chlorogenic, sinapic, and cinnamic acids. In contrast, ferulic acid contents in all breads with fermented cakes were lower than in the breads with unfermented cakes. Torreggiani et al. [40] and Novotni et al. [41] also observed that when raw bread materials or bread dough are submitted to lactic acid fermentation, the amount of free, easily available phenolic acids increased. The increase in caffeic, chlorogenic, sinapic, and syringic acid suggests the release or transformation of phenols by microbial enzymes during the fermentation process. The reduction in ferulic acid content in the breads with fermented cakes (i.e., with black cumin, camelina, and milk thistle cakes—ferulic acid content decreased from 120.40 to 89.60 mg/kg d.m., from 130.53 to 107.67 mg/kg d.m., and from 119.70 to 97.87 mg/kg d.m., respectively) likely resulted from biotransformation of this acid. Lactic acid bacteria primarily decarboxylate ferulic acid to 4-vinylguaiacol, while yeasts can additionally reduce and demethylate this compound to vanillin and ethylguaiacol. Studies by Boudaoud et al. [42] demonstrated that in the sourdough environment, *S. cerevisiae* yeasts convert ferulic acid primarily to 4-vinylguaiacol, while *L. plantarum* produces both 4-vinylguaiacol and dihydroferulic acid. 4-vinylguaiacol can be further reduced by reductase enzymes to 4-ethylguaiacol, a compound with aromatic properties. Some strains (e.g., *L. plantarum*) can also produce vanillin and vanillic acid by oxidation of part of the side chain. The next step may be the demethylation of these compounds to protocatechuic acid, which is further degraded under aerobic conditions by dioxygenases. Key enzymes playing a role in this transformation could be decarboxylases (PAD1, FDC1 in *S. cerevisiae*; PDC2 in *L. plantarum*) and phenolic acid reductases (hcrAB, especially hcrB in *L. plantarum*) [42].

The contents of the remaining analyzed bioactive compounds, such as thymoquinone (the bread with black cumin cake addition), silymarin (the bread with milk thistle cake addition), and carotenoids (the bread with camelina cake addition), in the breads with the addition of fermented cake were lower than the breads with unfermented cake addition.

It should be mentioned that the changes in phenolic compound content of breads containing fermented and unfermented cakes may result not only from the cakes’ fermentation process but also from thermal cleavage of bonds with polysaccharides and proteins, and structural transformations of phenolic compounds during bread production. Furthermore, baking-induced modifications of the bread matrix, such as starch gelatinization and cell wall degradation, may also increase the solubility and extractability of phenolic compounds, thus facilitating their chromatographic identification [43].

### 2.5. Fatty Acid Profile in Bread Containing Unfermented and Fermented Oilseed Cakes

Of all the fatty acids identified in the wheat bread (Table 5), the most abundant were linoleic acid (C18:2), oleic acid (C18:1), and palmitic acid (C16:0), which results from the high content of these acids in wheat flour, the main ingredient of the bread studied. In wheat bread, the share of SFAs was 29.35%, MUFAs 32.24%, and PUFAs 38.40%. The addition of cakes modified the fatty acid composition of the bread to varying degrees.

The greatest differences were observed in the case of the addition of unfermented black cumin cakes. Their addition resulted in a significant increase in the PUFA content (45.64%) and a decrease in the SFA (24.56%) and MUFA (29.80%) content. The addition of the remaining unfermented cakes only slightly modified the proportions of these three fatty acid groups. It is noteworthy that the presence of C18:3 n-3 (α-linolenic acid) was noted in the bread containing camelina cake, which constituted approximately 3% of all fatty acids. In bread with this addition, the higher PUFA/SFA ratio was also observed.

The results are in agreement with the study of Ivanova et al. [44]. They showed a significant decrease in SFA content and an increase in MUFA and PUFA in the case of wheat bread with the addition of chestnut, pumpkin seed, and rosehip flours (5% and 10%) compared to the control wheat bread. Osuna et al. [45] also reported the increase in PUFA/SFA ratio for the breads supplemented (5–15%) with flaxseed and soybean flours. The higher PUFA/SFA ratio improves wheat bread nutritional quality (a more positive effect on cardiovascular health), but it could also be a challenge due to the lower oxidation stability of bread lipids [27]. Analyzing the effect of camelina cake fermentation on the fatty acid profile in the bread made with it, it was found that the PUFA share was lower in the bread with fermented cake than in the bread with unfermented cake. Similar changes were observed when analyzing the unfermented and fermented cakes (Table 2).

## 3. Materials and Methods

### 3.1. Material

#### 3.1.1. Preparation of Cakes and Fermentation

The black cumin (*Nigella sativa* L.), camelina (*Camelina sativa* L.), rvening primrose (*Oenothera biennis* L.), and milk thistle (*Oenothera biennis* L.) cakes came from a producer of cold-pressed oil (Semco Sp. Z o. o. Sp.k., Szamotuły, Poland) and were used as the raw material in our experiment. The chemical composition of the raw material is presented in Table 6.

The cakes were ground with an IKA M20 (Ika, Staufen im Breisgau, Germany) laboratory grinder. The granulation of the raw materials was below 500 μm. Grinded samples were divided into two parts. One of them was analyzed directly and the other was fermented by using commercial starter culture of *Lactiplantibacillus plantarum* (Biochem s.r.l, Roma, Italy) according to Makowska et al. [12]. The amount of 1 g of starter culture was suspended in 400 mL of water (temperature 35 °C) containing 6 g of sucrose. The 200 g of ground cake was mixed with prepared starter culture suspension, covered, and put in a fermentation chamber (37 °C).

**Figure 1:**
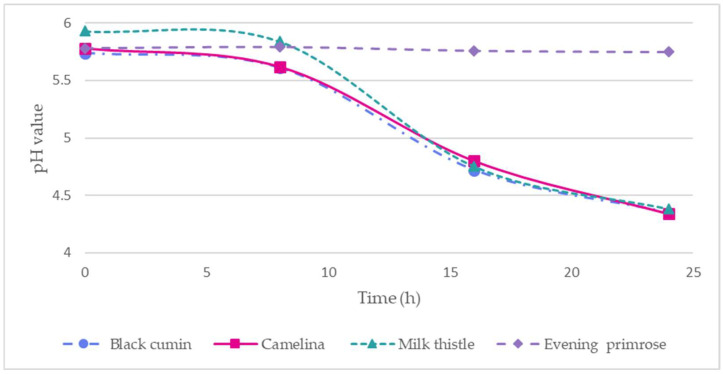
The pH changes in black cumin, camelina, milk thistle, and evening primrose cakes during fermentation time (h) with *L. plantarum* bacteria.

The fermentation process was monitored based on changes in the pH of the fermented medium. After 24 h of fermentation, the pH reached 4.3–4.4. The changes in the pH of the cakes during fermentation are shown in Figure 1.

After 24 h fermentation, the samples were first frozen at −24 °C and then freeze-dried using Christ Alpha 2–4 LD plus (Martin Christ Gefriertrocknungsanlagen GmbH, Osterode am Harz, Germany) at a pressure of 0.1 mbar and −20 °C for 48 h. All samples were stored in frozen conditions. The pictures of unfermented and fermented cakes are shown in the Supplementary Material (Figure S1).

The freeze-dried fermented cakes were used for further analysis and model bread preparation. The only exception was evening primrose cake, which under the above conditions showed no change in pH over 24 or even 48 h, indicating that *L. plantarum* bacteria did not ferment under these conditions.

#### 3.1.2. Preparation of Model Wheat Bread

Wheat flour 650 type (FPHU “ZŁOTY KŁOS’’ Józef Jurkiewicz, Wodzisław Śląski, Poland), fresh yeast, and salt were purchased by local shops. The bread dough was prepared using the single-phase method. All dough compounds (500 g of wheat flour, 15 g of yeast, and 7.5 g of salt and water) were mixed in a KitchenAid mixer (model 5KPM5EWH, KitchenAid Ariston, Benton Harbor, MI, USA) for 5 min. The dough was the control sample. In the experiment, part of the wheat flour was replaced with unfermented or fermented cakes at a quantity of 9%. The substitution amount was adopted based on preliminary research. The amount of water needed to prepare the dough was determined based on farinograph analysis (the amount of water needed to obtain the dough consistency of 500 BU). Next, the dough was placed in the fermentation chamber at 37 °C and a relative humidity of 75% (RH) for 60 min. After 30 min. the dough was punched. Then, it was divided into pieces of equal mass, removed into molds, and placed in a fermentation chamber for proofing (approximately 20 min). The loaves were baked in a baker’s oven (MIWE Michael Wenz GmbH, Arnstein, Germany) at 210 °C for 30 min. The bread was allowed to cool for 2 h at 20 °C; then, it was weighed and analyzed. Samples intended for analysis of bioactive compounds and fatty acids were freeze-dried, crushed, and stored in tight plastic bags until analysis. The pictures of the breads are presented in the Supplementary Material (Figure S2).

### 3.2. Methods

#### 3.2.1. Bioactive Compounds Content in Cakes and Breads

##### Determination of Flavonoids and Phenolic Acids Content

Phenolic acids and flavonoids were determined after subjecting the samples to successive alkaline and acid hydrolysis [46]. The extracts were analyzed using an Aquity UPLC H-Class system coupled with an Acquity PDA detector (Waters, Milford, MA, USA). Chromatographic separation was carried out on an Acquity UPLC^®^ BEH C18 column (100 mm × 2.1 mm, 1.7 μm; Waters, Wexford, Ireland). A gradient elution program was applied with the following mobile phases: solvent A, acetonitrile containing 0.1% formic acid, and solvent B, an aqueous 1% formic acid solution (pH 2). Quantification of phenolic compounds was performed using an external standard, with detection at 320 nm and 280 nm. Analytes were identified by comparing their retention times with those of reference standards and by spiking samples with known amounts of external standards, and the concentrations were expressed as mg per kg dry matter (d.m.) of sample.

##### Determination of Silymarin and Thymoquinone Content

Methanol extracts were used for silymarin and thymoquinone determination. The analysis was carried out using an Acquity UPLC system (Waters, USA) equipped with a Waters Acquity PDA detector (Waters, USA). Chromatographic separation was performed on an Acquity UPLC^®^ BEH C18 column (100 mm × 2.1 mm, 1.7 μm particle size) (Waters, Ireland). The elution system consisted of solvent A—0.1% phosphoric acid in water (*v*/*v*), and solvent B—acetonitrile (Merck, Darmstadt, Germany). A linear gradient elution was applied at a constant flow rate of 0.4 mL/min. The total run time was 10 min. The column was thermostated at 35 °C, and the samples were maintained at 10 °C before injection. The injection volume was 2 μL. Chromatographic detection was carried out at λ = 288 nm. Compound identification was based on retention times and UV spectra in the range of 200–400 nm, compared to authenticated standards of silybin A/B, isosilybin A/B, silychristin, silydianin, and taxifolin (Sigma-Aldrich, St. Louis, MO, USA) [47]. For thymoquinone, detection was performed at a wavelength of 254 nm. Quantitative results were expressed as the total sum of silymarin components and thymoquinone, recalculated as mg/kg d.m. of test material.

##### Determination of Carotenoid Content

Carotenoids were extracted from cake samples using a saponification procedure [46]. Lutein, zeaxanthin, and β-carotene were quantified on an Acquity UPLC system (Waters, USA) equipped with an Acquity PDA detector (Waters, USA). Chromatographic separation was achieved on an Acquity UPLC^®^ BEH C18 column (100 mm × 2.1 mm, 1.7 μm; Waters, Ireland). The mobile phase consisted of methanol (solvent A), water, and tert-butyl methyl ether (TBME) (solvent B), delivered in a gradient mode at a flow rate of 0.4 mL/min. The column and autosampler were maintained at 30 °C and 10 °C, respectively. Detection was performed at 445 nm, and carotenoid contents were expressed as mg per kg d.m. of sample material.

#### 3.2.2. Fatty Acid Profile (FAME) Analysis in Cakes and Breads

Fatty acids were extracted by a method described by Przybylska-Balcerek et al. [46] and analyzed by an Aquity H class UPLC system equipped with a Waters Acquity PDA detector (Waters, USA). Chromatographic separation was performed on an Acquity UPLC^®^ BEH C18 column (150 mm × 2.1 mm, particle size 1.7 μm) (Waters, Ireland). The elution was carried out in gradient using the following mobile phase composition: A—acetonitrile; B—2-propanol, at a flow of flow 0.17 mL/min.

#### 3.2.3. Bread Physical Characteristics

##### Bread Volume

The volume of bread was determined according to AACC 10-05.01 standard procedure [48] and calculated in mL/100 g of bread.

##### Bread Crumb and Crust Color Analysis

A Konica Minolta CR-410 colorimeter (Konica Minolta Sensing Inc., Tokyo, Japan) was employed to measure the color of both the bread crumb and crust. The measurements were expressed in the CIE Lab* color space, where L* represents lightness, a* indicates the red–green coordinate, and b* denotes the yellow–blue coordinate. The color difference between breads containing unfermented and fermented cakes was calculated using the appropriate color difference equation as previously described [49]:

$$∆E=\sqrt{{∆L}^{2}+{∆a}^{2}+{∆b}^{2}}$$

Additionally, whiteness index (WI) and browning index (BI) values were calculated for bread crumb and crust, respectively, according to Djordjević et al. [38].

##### Bread Texture Analysis

Texture profile analysis of bread was performed using a TA.XTplus texture analyzer (Stable Micro System Co., Ltd., Surrey, UK) equipped with a 5 kg load cell. Slices of bread, each 25 mm thick, were subjected to two compression cycles with a cylindrical probe having a 36 mm diameter. Test settings for the instrument included a pre-test speed, test speed, and post-test speed of 5.0 mm/s, with the samples compressed to 40% strain. A 5 s interval was maintained between the two compressions. The parameters assessed during the procedure included hardness, springiness, cohesiveness, chewiness, and resilience. Each measurement was repeated five times.

#### 3.2.4. Statistical Analysis

Data on the properties of cakes were subjected to one-way analysis of variance. In contrast, the results concerning bread were subjected to two-way analysis of variance (ANOVA) (Statistica 13.3, TIBCO Software Inc., Palo Alto, CA, USA) followed by post hoc Tukey’s HSD test at *p* ≤ 0.05 significance. Correlation coefficients between dietary fiber content and texture parameters were calculated.

## 4. Conclusions

This study demonstrates that lactic acid fermentation with *Lactiplantibacillus plantarum* represents an effective biotechnological approach for valorizing oilseed cakes and modulating their bioactive compound profiles. Fermentation significantly modified the phytochemical profiles of the cakes. Camelina cake showed the richest flavonoid content (36.14 mg/kg d.m. unfermented, 46.94 mg/kg d.m. fermented) and highest phenolic acid concentration (1149.60 mg/kg d.m. unfermented, increasing ~30% after fermentation), with sinapic acid as the dominant compound. Black cumin cake contained 1150 mg/kg d.m. thymoquinone, which decreased by 10% after fermentation, while 4-hydroxybenzoic acid increased eightfold. Milk thistle cake silymarin content (30.53 mg/kg) decreased approximately 10% post-fermentation. Carotenoids in camelina cake (β-carotene 30.50 mg/kg d.m., lutein 3.55 mg/kg d.m.) declined by 30% after fermentation. Fermentation increased saturated (SFAs) and monounsaturated fatty acids (MUFAs) while reducing polyunsaturated fatty acids (PUFAs) in all cakes.

Incorporation of fermented cakes at 9% substitution level in wheat bread successfully enhanced bioactive compound content, particularly in the camelina-fortified bread, which exhibited 15% higher flavonoid levels compared to unfermented variants. However, fermented cakes adversely affected bread’s physical properties, causing a reduction in specific volume and an increase in hardness, gumminess, and chewiness. To the best of the authors’ knowledge, the data on the profile of individual phenolic compound contents in breads with the addition of lactic fermented cakes are reported for the first time. These findings indicate that lactic fermentation effectively enriches oilseed cakes with bioactive compounds; however, optimization of fermentation parameters and dough formulation is crucial to achieve acceptable bread quality while maintaining nutritional benefits.

However, the same limitations of this study should be acknowledged. In the study, only a single level of wheat flour replacement with selected oilseed cakes (9%) was evaluated, which limits the ability to generalize the results and does not allow for determining the optimal levels of additives for different technological and sensory purposes. Moreover, lactic acid fermentation was carried out using one commercial *L. plantarum* starter under fixed process conditions. Future research should therefore be conducted to explore various substitution levels and combinations of oilseed cakes, including diverse LAB strains and fermentation regimes tailored to specific substrates. The mechanisms underlying the observed changes in the content of bioactive compounds resulting from fermentation (including bacterial enzymes activity) also require clarification.

## Figures and Tables

**Table 1:** Bioactive compound content (mg/kg d.m.) in the unfermented (UF) and the fermented (F) oilseed cakes.

Compounds	Black Cumin		Camelina		Milk Thistle		Evening Primrose
	UF	F	UF	F	UF	F	UF
**Flavonoids**							
**Apigenin**	nd	nd	11.22 ± 0.04 ^b^	14.59 ± 0.04 ^a^	nd	nd	nd
**Catechin**	nd	nd	1.12 ± 0.02 ^b^	1.47 ± 0.02 ^a^	nd	nd	0.10 ± 0.01 ^c^
**Kaempferol**	nd	nd	2.65 ± 0.02 ^b^	3.41 ± 0.03 ^a^	nd	nd	0.60 ± 0.06 ^c^
**Luteolin**	nd	nd	3.5 ± 0.02 ^b^	4.56 ± 0.02 ^a^	nd	nd	0.05 ± 0.01 ^c^
**Naringenin**	nd	nd	3.12 ± 0.03 ^b^	4.03 ± 0.02 ^a^	nd	nd	0.02 ± 0.00 ^c^
**Quercetin**	nd	nd	6.54 ± 0.02 ^b^	8.56 ± 0.05 ^a^	nd	nd	0.01 ± 0.00 ^c^
**Rutin**	nd	nd	6.22 ± 0.02 ^b^	8.08 ± 0.03 ^a^	0.03 ± 0.01 ^d^	0.04 ± 0.01 ^d^	4.80 ± 0.10 ^c^
**Vitexin**	nd	nd	3.26 ± 0.01 ^c^	4.19 ± 0.08 ^b^	0.08 ± 0.01 ^d^	0.10 ± 0.01 ^d^	7.50 ± 0.02 ^a^
**SUM**	-	-	36.14	46.94	0.11	0.13	12.58
**Phenolic acids**							
**4-hydroxybenzoic**	0.10 ± 0.01 ^c^	0.81 ± 0.59 ^c^	35.60 ± 0.07 ^b^	46.29 ± 0.07 ^a^	nd	nd	nd
**Caffeic**	nd	nd	132.70 ± 0.45 ^b^	171.88 ± 0.81 ^a^	nd	nd	5.00 ± 0.24 ^c^
**Chlorogenic**	nd	nd	50.28 ± 0.15 ^b^	65.21 ± 0.12 ^a^	0.05 ± 0.00 ^d^	0.07 ± 0.01 ^d^	3.20 ± 0.09 ^c^
**Ferulic**	nd	nd	82.32 ± 0.14 ^b^	107.41 ± 0.39 ^a^	nd	nd	14.80 ± 0.14 ^c^
**Gallic**	nd	nd	12.35 ± 0.06 ^c^	16.11 ± 0.09 ^b^	nd	nd	63.40 ± 0.05 ^a^
***p*** **-Coumaric**	0.09 ± 0.00 ^d^	0.12 ± 0.00 ^d^	19.35 ± 0.06 ^b^	25.17 ± 0.04 ^a^	0.11 ± 0.00 ^d^	0.15 ± 0.01 ^d^	7.35 ± 0.00 ^c^
**Protocatechuic**	nd	nd	9.33 ± 0.02 ^b^	12.20 ± 0.06 ^a^	nd	nd	nd
**Sinapic**	nd	nd	652.22 ± 0.52 ^b^	848.18 ± 0.14 ^a^	nd	nd	nd
**Syringic**	nd	nd	21.21 ± 0.09 ^b^	27.66 ± 0.13 ^a^	nd	nd	nd
**Cinnamic**	nd	nd	132.97 ± 0.64 ^b^	172.71 ± 0.61 ^a^	nd	nd	nd
**Vanillic**	nd	nd	1.25 ± 0.02 ^b^	1.64 ± 0.02 ^a^	nd	nd	nd
**SUM**	0.19	0.93	1149.60	1494.45	0.16	0.22	93.75
**Others**							
**Thymoquinone**	11,490.00 ± 11.14 ^a^	10,345.67 ± 5.13 ^b^	nd	nd	nd	nd	nd
**Silymarins**	nd	nd	nd	nd	30.53 ± 0.10 ^a^	27.50 ± 0.15 ^b^	nd
**Lutein**	nd	nd	3.55 ± 0.02 ^a^	2.45 ± 0.04 ^b^	nd	nd	nd
**Zeaxanthin**	nd	nd	0.05 ± 0.01 ^a^	0.03 ± 0.01 ^a^	nd	nd	nd
**β-carotene**	nd	nd	30.50 ± 0.08 ^a^	21.38 ± 0.05 ^b^	nd	nd	nd

nd—not detected. Values (mean ± SD of three replicates) bearing different superscripts are statistically significantly different (*p* ≤ 0.05).

**Table 2:** Fatty acid composition of the unfermented (UF) and fermented (F) cakes.

Fatty Acid	Black Cumin		Camelina		Milk Thistle		Evening Primrose
	UF	F	UF	F	UF	F	UF
**C14:0**	0.00	0.11 ± 0.01 ^c^	0.29 ± 0.01 ^b^	0.39 ± 0.02 ^a^	0.00	0.10 ± 0.01 ^c^	0.00
**C15:0**	0.00	0.51 ± 0,01 ^b^	0.00	0.46 ± 0.05 ^c^	0.00	0.60 ± 0.01 ^a^	0.00
**C16:0**	16.70 ± 0.03 ^c^	18.60 ± 0.10 ^a^	3.50 ± 0.01 ^f^	4.53 ± 0.06 ^e^	15.60 ± 0.02 ^d^	17.13 ± 0.15 ^b^	7.80 ± 0.01 ^e^
**C16:1**	0.00	0.10 ± 0.00 ^a^	0.09 ± 0.01 ^a^	0.17 ± 0.06 ^a^	0.00	0.13 ± 0.06 ^a^	0.00
**C17:0**	0.00	0.50 ± 0.01 ^ab^	0.00	0.48 ± 0.03 ^b^	0.00	0.51 ± 0.01 ^a^	0.00
**C18:0**	5.30 ± 0.01 ^b^	6.07 ± 0.06 ^a^	2.39 ± 0.03 ^e^	3.45 ± 0.05 ^d^	3.50 ± 0.05 ^d^	4.51 ± 0.02 ^c^	1.80 ± 0.01 ^f^
**C18:1**	17.60 ± 0.02 ^c^	20.74 ± 0.23 ^a^	14.46 ± 0.05 ^d^	18.10 ± 0.19 ^b^	12.40 ± 0.01 ^d^	14.78 ± 0.19 ^d^	6.70 ± 0.03 ^f^
**C18:2**	60.40 ± 0.01 ^c^	52.13 ± 0.15 ^e^	18.34 ± 0.04 ^f^	15.50 ± 0.10 ^g^	68.50 ± 0.07 ^b^	61.70 ± 0.10 ^c^	74.90 ± 0.02 ^a^
**C18:3 n-6**	0.00	0.00	0.00	0.00	0.00	0.00	8.70 ± 0.02 ^a^
**C18:3 n-3**	0.00	0.00	36.84 ± 0.06 ^a^	31.40 ± 0.10 ^b^	0.00	0.00	0.10 ± 0.02 ^c^
**C20:0**	0.00	0.17 ± 0.06 ^c^	0.89 ± 0.05 ^b^	1.10 ± 0.10 ^a^	0.00	0.23 ± 0.06 ^c^	0.00
**C20:1**	0.00	0.00	18.75 ± 0.01 ^b^	19.50 ± 0.10 ^a^	0.00	0.00	0.00
**C20:2**	0.00	0.00	0.49 ± 0.01 ^a^	0.53 ± 0.06 ^a^	0.00	0.00	0.00
**C22:1**	0.00	0.00	3.27 ± 0.06 ^a^	3.30 ± 0.01 ^a^	0.00	0.00	0.00
**C24:0**	0.00	1.07 ± 0.06 ^a^	0.39 ± 0.01 ^c^	0.60 ± 0.01 ^b^	0.00	0.29 ± 0.01 ^d^	0.00
**C24:1**	0.00	0.00	0.30 ± 0.02 ^b^	0.49 ± 0.02 ^a^	0.00	0.00	0.00
**∑SFAs**	22.00	27.03	7.46	11.01	19.10	23.38	9.60
**∑MUFAs**	17.60	20.84	36.87	41.56	12.40	14.91	6.70
**∑PUFAs**	60.40	52.13	55.67	47.43	68.50	61.70	83.70
**∑PUFA/∑SFA**	2.75	1.93	7.46	4.31	3.59	2.64	8.72
**SUM**	100	100	100	100	100	100	100

Values (mean ± SD of three replicates) bearing different superscripts are statistically significantly different (*p* ≤ 0.05).

**Table 3:** Physical characteristics of wheat bread and breads containing unfermented (UF) and fermented (F) oilseed cakes.

Characteristics	Wheat Bread Containing 9%						Wheat Bread
	Black Cumin Cake		Cameline Cake		Milk Thistle Cake		
	UF	F	UF	F	UF	F	
**Texture parameters**							
**Hardness (N)**	23.72 ± 1.11 ^bc^	28.99 ± 2.14 ^bc^	13.68 ± 1.38 ^de^	24.62 ± 1.45 ^c^	28.31 ± 3.18 ^a^	34.91 ± 1.94 ^a^	6.63 ± 1.29 ^f^
**Springiness (%)**	0.82 ± 0.03 ^e^	0.75 ± 0.07 ^e^	0.93 ± 0.02 ^abcd^	0.84 ± 0.03 ^abcd^	0.83 ± 0.07 ^e^	0.82 ± 0.04 ^a^	0.89 ± 0.05 ^abcde^
**Cohesiveness (-)**	0.76 ± 0.03 ^d^	0.73 ± 0.04 ^d^	0.87 ± 0.03 ^abc^	0.76 ± 0.03 ^a^	0.80 ± 0.03 ^cd^	0.78 ± 0.02 ^cd^	0.86 ± 0.02 ^ab^
**Gumminess (-)**	18.08 ± 1.40 ^c^	21.25 ± 1.09 ^c^	11.41 ± 0.91 ^d^	18.80 ± 1.06 ^c^	22.54 ± 1.70 ^a^	27.03 ± 1.78 ^a^	5.74 ± 1.17 ^f^
**Chewiness (-)**	14.83 ± 1.75 ^cd^	16.06 ± 2.07 ^c^	10.66 ± 0.88 ^ef^	15.85 ± 1.44 ^abc^	18.84 ± 2.74 ^ab^	22.16 ± 2.33 ^ab^	5.14 ± 1.14 ^g^
**Resilience (-)**	0.30 ± 0.01 ^h^	0.29 ± 0.03 ^h^	0.46 ± 0.03 ^bcde^	0.35 ± 0.02 ^abcd^	0.42 ± 0.02 ^efg^	0.39 ± 0.03 ^ab^	0.48 ± 0.03 ^abcde^
**Specific volume (mL/100 g)**	265 ± 3 ^ea^	186 ± 4 ^e^	233 ± 2 ^i^	226 ± 3 ^k^	242 ± 2 ^ghb^	180 ± 6 ^e^	238 ± 3 ^hi^
**Crumb Color**							
L*	30.1 ± 1.0 ^f^	53.0 ± 0.6 ^c^	49.6 ± 1.0 ^d^	56.1 ± 0.9 ^b^	47.2 ± 0.7 ^e^	56.3 ± 0.1 ^b^	70.6 ± 0.4 ^a^
a*	2.4 ± 0.0 ^d^	5.3 ± 0.1 ^a^	4.5 ± 0.0 ^b^	5.5 ± 0.3 ^a^	3.8 ± 0.3 ^c^	4.6 ± 0.1 ^b^	2.0 ± 0.1 ^e^
b*	0.8 ± 0.1 ^g^	11.1 ± 0.1 ^e^	12.7 ± 0.2 ^d^	17.4 ± 0.6 ^a^	10.5 ± 0.0 ^f^	15.5 ± 0.2 ^c^	16.6 ± 0.1 ^b^
**WI**	30.05	51.46	47.83	52.43	46.03	53.41	66.18
**ΔE**	-	11.8	-	8.1	-	10.4	-
**Crust Color**							
L*	32.3 ± 1.0 ^f^	35.7 ± 0.4 ^e^	44.0 ± 0.7 ^d^	55.7 ± 0.2 ^c^	44.7 ± 0.4 ^d^	59.4 ± 0.1 ^b^	63.9 ± 1.6 ^a^
a*	2.5 ± 0.0 ^f^	3.9 ± 0.2 ^e^	9.9 ± 0.6 ^a^	10.2 ± 0.2 ^a^	8.9 ± 0.0 ^b^	8.6 ± 0.1 ^c^	5.8 ± 0.7 ^d^
b*	3.0 ± 0.2 ^g^	5.0 ± 0.6 ^f^	15.0 ± 0.4 ^fd^	24.6 ± 0.2 ^b^	13.2 ± 0.1 ^e^	22.4 ± 0.1 ^c^	26.4 ± 0.5 ^a^
**BI**	21.91	29.69	66.78	80.99	57.78	66.83	68.80
**ΔE**	-	4.2	-	15.1	-	14.4	-

Values (mean ± SD of three replicates) bearing different superscripts are statistically significantly different (*p* ≤ 0.05).

**Table 4:** Bioactive compound content (mg/kg d.m.) in wheat bread and breads containing unfermented (UF) and fermented (F) oilseed cakes.

Compounds	Wheat Bread Containing 9% of						Wheat Bread
	Black Cumin Cake		Camelina Cake		Milk Thistle Cake		
	UF	F	UF	F	UF	F	
**Flavonoids**							
**Apigenin**	nd	nd	1.16 ± 0.03 ^a^	1.32 ± 0.01 ^b^	nd	nd	nd
**Catechin**	nd	nd	0.12 ± 0.02 ^a^	0.13 ± 0.00 ^a^	nd	nd	nd
**Kaempferol**	nd	nd	0.27 ± 0.01 ^b^	0.30 ± 0.00 ^a^	nd	nd	nd
**Luteolin**	nd	nd	0.36 ± 0.01 ^b^	0.42 ± 0.01 ^a^	nd	nd	nd
**Naringenin**	nd	nd	0.32 ± 0.02 ^b^	0.35 ± 0.01 ^a^	nd	nd	nd
**Quercetin**	nd	nd	0.66 ± 0.03 ^b^	0.76 ± 0.01 ^a^	nd	nd	nd
**Rutin**	nd	nd	0.63 ± 0.02 ^b^	0.72 ± 0.01 ^a^	nd	nd	nd
**Vitexin**	nd	nd	0.33 ± 0.03 ^a^	0.37 ± 0.01 ^a^	0.01 ± 0.00 ^c^	0.01 ± 0.00 ^c^	nd
**SUM**	nd	nd	3.85	4.37	0.01	0.01	nd
**Phenolic acids**							
**4-hydroxybenzoic**	0.01 ± 0.01 ^c^	0.01 ± 0.00 ^c^	3.53 ± 0.04 ^b^	4.08 ± 0.08 ^a^	nd	nd	nd
**Caffeic**	nd	nd	13.25 ± 0.04 ^b^	15.65 ± 0.30 ^a^	nd	nd	nd
**Chlorogenic**	nd	nd	5.03 ± 0.02 ^b^	5.88 ± 0.06 ^a^	0.01 ± 0.00 ^c^	0.01 ± 0.00 ^c^	nd
**Ferulic**	120.40 ± 0.20 ^bc^	89.60 ± 0.70 ^e^	130.53 ± 0.45 ^a^	107.67 ± 0.20 ^d^	119.70 ± 0.36 ^c^	97.87 ± 0.70 ^e^	121.23 ± 0.80 ^b^
**Gallic**	nd	nd	1.25 ± 0.03 ^b^	1.39 ± 0.03 ^a^	nd	nd	nd
***p*** **-Coumaric**	nd	nd	1.25 ±0.02 ^b^	1.39 ± 0.03 ^a^	nd	nd	nd
**Protocatechuic**	nd	nd	0.93 ± 0.02 ^b^	1.09 ± 0.02 ^a^	nd	nd	nd
**Sinapic**	nd	nd	65.26 ± 0.03 ^b^	76.32 ± 0.96 ^a^	nd	nd	nd
**Syringic**	nd	nd	2.15 ± 0.03 ^b^	2.48 ± 0.01 ^a^	nd	nd	nd
**Cinnamic**	nd	nd	13.23 ± 0.03 ^b^	15.14 ± 0.48 ^a^	nd	nd	nd
**Vanillic**	nd	nd	0.13 ± 0.01 ^a^	0.14 ± 0.00 ^a^	nd	nd	nd
**SUM**	120.41	89.61	236.53	231.25	119.71	97.88	121.23
**Others**							
**Thymoquinone**	1155.00 ± 3.61 ^a^	921.15 ± 12.69 ^b^	nd	nd	nd	nd	nd
**Silymarins**	nd	nd	nd	nd	2.75 ± 0.05 ^a^	2.64 ± 0.02 ^b^	nd
**Lutein**	nd	nd	0.34 ± 0.02 ^a^	0.22 ± 0.01 ^b^	nd	nd	nd
**Zeaxanthin**	nd	nd	0.01 ± 0.01 ^a^	nd	nd	nd	nd
**β-carotene**	nd	nd	3.07 ± 0.03 ^a^	1.88 ± 0.03 ^b^	nd	nd	nd

Values (mean ± SD of three replicates) bearing different superscripts are statistically significantly different (*p* ≤ 0.05).

**Table 5:** Composition (%) of fatty acids in wheat bread and the breads containing unfermented and fermented oilseed cakes.

Fatty Acid	Wheat Bread Containing						Wheat Bread
	Black Cumin Cake		Camelina Cake		Milk Thistle Cake		
	UF	F	UF	F	UF	F	
**C14:0**	0.19 ± 0.01 ^cd^	0.21 ± 0.02 ^ab^	0.23 ± 0.01 ^a^	0.23 ± 0.01 ^ab^	0.20 ± 0.02 ^bcd^	0.20 ± 0.01 ^abc^	0.19 ± 0.01 ^cd^
**C16:0**	19.40 ± 0.10 ^b^	19.90 ± 0.10 ^a^	18.83 ± 0.35 ^b^	19.10 ± 0.06 ^b^	19.23 ± 0.15 ^b^	19.50 ± 0.10 ^b^	21.00 ± 1.00 ^a^
**C16:1**	0.55 ± 0.04 ^ab^	0.50 ± 0.00 ^abc^	0.53 ± 0.02 ^abc^	0.55 ± 0.00 ^a^	0.52 ± 0.03 ^abc^	0.50 ± 0.00 ^abc^	0.47 ± 0.06 ^bc^
**C18:0**	3.67 ± 0.06 ^b^	10.20 ± 0.06 ^a^	9.22 ± 0.10 ^a^	9.02 ± 0.04 ^a^	9.60 ± 0.00 ^a^	9.90 ± 0.05 ^a^	9.90 ± 0.10 ^a^
**C18:1**	26.87 ± 5.69 ^a^	31.09 ± 0.06 ^a^	26.53 ± 0.25 ^a^	30.30 ± 0.10 ^a^	29.40 ± 0.10 ^a^	30.70 ± 0.10 ^a^	30.10 ± 0.10 ^a^
**C18:2**	45.36 ± 9.41 ^a^	33.80 ± 0.41 ^a^	34.87 ± 0.59 ^a^	31.20 ± 0.39 ^a^	37.18 ± 0.23 ^a^	34.90 ± 21.80 ^a^	33.88 ± 1.19 ^a^
**C18:3**	0.00	0.00	0.00	0.00	0.00	0.00	0.00
**C18:3 n-3**	0.00	0.00	3.37 ± 0.12 ^a^	3.10 ± 0.06 ^b^	0.00	0.00	0.00
**C20:0**	0.81 ± 0.01 ^c^	0.97 ± 0.06 ^abc^	0.92 ± 0.01 ^bc^	1.03 ± 0.06 ^ab^	0.93 ± 0.01 ^bc^	1.10 ± 0.10 ^a^	1.10 ± 0.10 ^a^
**C20:1**	1.93 ± 0.06 ^bc^	2.17 ± 0.12 ^b^	3.80 ± 0.10 ^a^	3.90 ± 0.06 ^a^	1.83 ± 0.06 ^c^	2.00 ± 0.10 b^b^	2.03 ± 0.06 ^bc^
**C20:2**	0.28 ± 0.01 ^a^	0.30 ± 0.01 ^a^	0.32 ± 0.01 ^a^	0.30 ± 0.02 ^a^	0.20 ± 0.01 ^a^	0.30 ± 18.30 ^a^	0.31 ± 0.02 ^a^
**C22:1**	0.00	0.00	0.31 ± 0.02 ^a^	0.30 ± 0.01 ^a^	0.00	0.00	0.00
**C24:0**	0.49 ± 0.01 ^bc^	0.50 ± 0.01 ^bc^	0.55 ± 0.01 ^a^	0.50 ± 0.01 ^b^	0.48 ± 0.01 ^c^	0.50 ± 0.01 ^b^	0.51 ± 0.01 ^b^
**C24:1**	0.45 ± 0.03 ^b^	0.40 ± 0.01 ^c^	0.52 ± 0.01 ^a^	0.50 ± 0.01 ^a^	0.43 ± 0.01 ^bc^	0.40 ± 0.01 ^c^	0.51 ± 0.01 ^a^
**∑SFAs**	24.56	31.81	29.75	29.85	30.44	31.20	29.35
**∑MUFAs**	29.80	34.09	31.69	35.55	32.18	33.60	32.24
**∑PUFAs**	45.64	34.10	38.56	34.60	37.38	35.20	38.40
**∑PUFA/∑SFA**	1.86	1.07	1.30	1.16	1.23	1.13	1.31
**SUM**	100.00	100.00	100	100.00	100.00	100.00	100.00

Values (mean ± SD of three replicates) bearing different superscripts are statistically significantly different (*p* ≤ 0.05).

**Table 6:** Chemical composition of the analyzed cakes.

Type of Cake	Compounds (g/100 g d.m.)						Dry Matter
	Protein	Total Fat	Ash	IDF	SDF	TDF	
**Black** **cumin**	28.64 ± 1.04	17.65 ± 0.41	6.67 ± 0.19	27.63 ± 0.40	5.15 ± 0.46	32.78	92.42 ± 0.09
**Camelina**	33.60 ± 1.23	14.04 ± 0.28	6.88 ± 0.04	31.50 ± 4.77	10.16 ± 1.68	41.66	89.35 ± 0.02
**Evening primrose**	21.91 ± 0.52	10.22 ± 0.16	7.04 ± 0.09	58.60 ± 0.39	0.56 ± 0.20	59.16	92.15 ± 0.05
**Milk thistle**	20.09 ± 0.15	8.24 ± 0.12	6.99 ± 0.06	50.94 ± 0.06	3.88 ± 0.50	54.82	93.19 ± 0.29

Values (mean ± SD).

## Data Availability

Data are contained within the article or the Supplementary Material.

## Supplementary Materials

The following supporting information can be downloaded at https://www.mdpi.com/article/10.3390/molecules31010015/s1; Figure S1: photos of unfermented (UF) and fermented (F) cakes used in the experiment; Figure S2: photos of wheat breads containing unfermented (UF) and fermented (F) cakes (9% substitution of wheat flour).
