# Supplementary material for: Oilseed By-Products Valorization Using Lactic Acid Fermentation: Nutritional and Technological Aspects of Applications in Wheat Bread
Source: Molecules. 2025 Dec 20;31(1):15. doi: 10.3390/molecules31010015 (PMC12786963; doi:10.3390/molecules31010015)
Supplement: Supplementary file 1 [file molecules-31-00015-s001.zip › molecules-4012148-supplementary.pdf]

Oilseed By-Products Valorization Using Lactic Acid Fermentation: Nutritional and Technological Aspects of Applications in Wheat Bread

Jakub Roman Królak, Agnieszka Makowska* , Katarzyna Waszkowiak*, Kamila Myszka, Kinga Stuper-Szablewska , Anna Przybylska-Balcerek and Katarzyna Rzyska-Szczupak

MOLECULES

Supplementary Materials

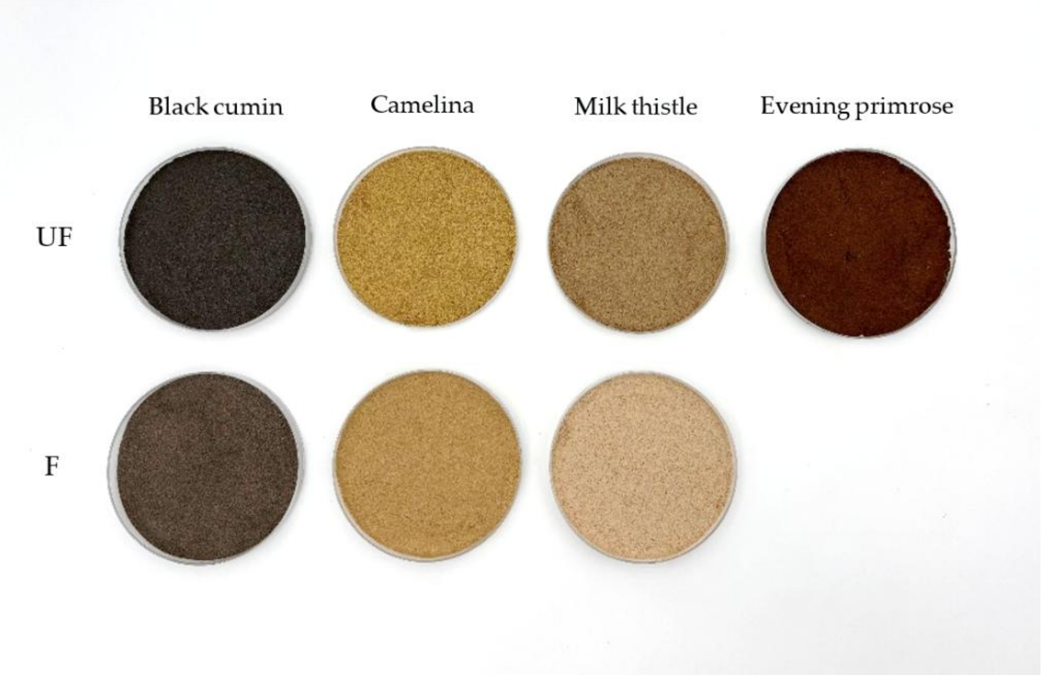

Figure S1. Photos of unfermented (UF) and fermented (F) cakes used in the experiment.

Oilseed By-Products Valorization Using Lactic Acid Fermentation: Nutritional and Technological Aspects of Applications in Wheat Bread

Jakub Roman Królak, Agnieszka Makowska*, Katarzyna Waszkowiak*, Kamila Myszka, Kinga Stuper-Szablewska, Anna Przybylska-Balcerek and Katarzyna Rzyska-Szczupak

MOLECULES

Supplementary Materials

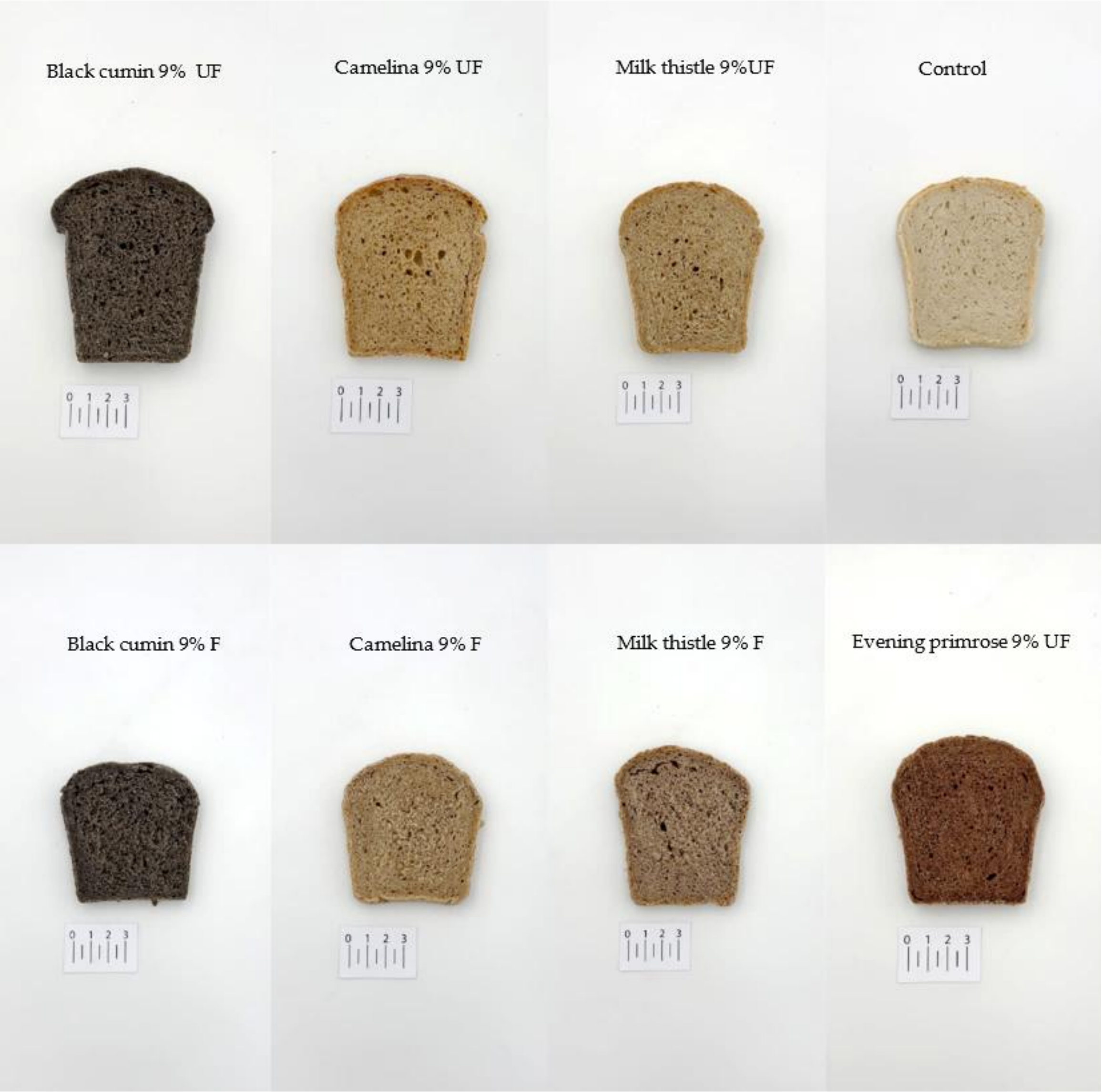

Figure S2. Photos of wheat breads containing unfermented (UF) and fermented (F) cakes (9% substitution of wheat flour).
